# Using new technologies to promote weight management: a randomised controlled trial study protocol

**DOI:** 10.1186/s12889-015-1849-4

**Published:** 2015-05-27

**Authors:** Monica Jane, Jonathan Foster, Martin Hagger, Sebely Pal

**Affiliations:** School of Public Health, Faculty of Health Sciences, Curtin University, GPO Box U1987, Perth, Western Australia; School of Psychology and Speech Pathology, Faculty of Health Sciences, Curtin University, GPO Box U1987, Perth, Western Australia

**Keywords:** Overweight, Obesity, Weight management, Social media, Facebook, Health promotion, Dietary intake, Physical activity, Social support, Total Wellbeing Diet

## Abstract

**Background:**

Over the last three decades, overweight and obesity and the associated health consequences have become global public health priorities. Methods that have been tried to address this problem have not had the desired impact, suggesting that other approaches need to be considered. One of the lessons learned throughout these attempts is that permanent weight loss requires sustained dietary and lifestyle changes, yet adherence to weight management programs has often been noted as one of the biggest challenges. This trial aims to address this issue by examining whether social media, as a potential health promotion tool, will improve adherence to a weight management program. To test the effectiveness of this measure, the designated program will be delivered via the popular social networking site Facebook, and compared to a standard delivery method that provides exactly the same content but which is communicated through a pamphlet. The trial will be conducted over a period of twelve weeks, with a twelve week follow-up. Although weight loss is expected, this study will specifically investigate the effectiveness of social media as a program delivery method. The program utilised will be one that has already been proven to achieve weight loss, namely *The CSIRO Total Wellbeing Diet*.

**Methods/design:**

This project will be conducted as a 3-arm randomised controlled trial. One hundred and twenty participants will be recruited from the Perth community, and will be randomly assigned to one of the following three groups: the Facebook group, the pamphlet group, or a control group. The Facebook Group will receive the weight management program delivered via a closed group in Facebook, the Pamphlet Group will be given the same weight management program presented in a booklet, and the Control Group will follow the Australian Dietary Guidelines and the National Physical Activity Guidelines for Adults as usual care. Change in weight, body composition and waist circumference will be initial indicators of adherence to the program. Secondary outcome measures will be blood glucose, insulin, blood pressure, arterial stiffness, physical activity, eating behaviour, mental well-being (stress, anxiety, and depression), social support, self-control, self-efficacy, Facebook activity, and program evaluation.

**Discussion:**

It is expected that this trial will support the use of social media - a source of social support and information sharing - as a delivery method for weight management programs, enhancing the reduction in weight expected from dietary and physical activity changes. Facebook is a popular, easy to access and cost-effective online platform that can be used to assist the formation of social groups, and could be translated into health promotion practice relatively easily. It is anticipated in the context of the predicted findings that social media will provide an invaluable resource for health professionals and patients alike.

**Trial registration:**

Australian New Zealand Clinical Trials Register (ANZCTR): ACTRN12614000536662. Date registered: 21 May 2014.

**Electronic supplementary material:**

The online version of this article (doi:10.1186/s12889-015-1849-4) contains supplementary material, which is available to authorized users.

## Background

World-wide rates of overweight and obesity continue to rise, despite the growing awareness of the importance of this issue in recent years among health professionals [1–4] as well as the general public [5]. Indeed, there is a widespread lack of acceptance of obesity in the general community, perhaps relating more to the physical appearance of people with obesity rather than the associated health risks [5, 6]. It is also well-established that being socioeconomically disadvantaged increases the risk of overweight and obesity [7]. The health consequences of excessive weight gain include an increased risk of the metabolic syndrome, and such chronic diseases as diabetes, cardiovascular disease, obstructive sleep apnoea and some cancers, all potentially leading to increased mortality [2, 4]. The psychosocial consequences of obesity include stigmatisation in the workplace, compromised health care and personal relationships [5, 8] and reduced quality of life [4, 8].

A review of the relevant literature has revealed that an energy-restricted, low fat, high protein diet assists with weight loss [9, 10] and the reduction of cardiometabolic risk factors [11]. It also increases satiety [12], which is an important factor in dietary compliance [13], and assists with weight loss maintenance [12]. Increasing physical activity has also been shown to improve cardiometabolic risk factors in both short and long- term trials [14, 15]. According to the National Physical Activity Guidelines for Adults, thirty minutes of moderate physical activity (preferably taken every day) is required to promote health [16].

The CSIRO Total Wellbeing Diet (TWD), developed by the Commonwealth Scientific and Industrial Research Organisation (CSIRO) [17], is an energy-reduced, low fat, higher protein diet that meets the Australian nutrient reference values for daily intake [18], promotes a minimum thirty minutes of moderate physical activity per day, and has been extensively researched [9, 11, 19, 20]. In Book 2 of the TWD, the use of a pedometer is recommended to help individuals meet the suggested physical activity target [17]. Pal et al. have shown that setting a goal of 10,000 steps per day results in greater improvements to physical activity levels than the 30-min-a-day target [21]. This weight management program, along with the support of a dietitian, has been shown to cause significant improvements in cardiovascular disease risk biomarkers in overweight and obese individuals and weight loss of up to 10 kg after twelve weeks [11, 20]. However a mean weight loss of 5 kg has been reported by individuals following the TWD Book alone [22].

While weight loss can reduce the health risks associated with obesity [12, 23], many dieters have difficulty adhering to weight loss programs [24] or maintaining long-term weight loss [12, 23]. This can contribute to a sense of personal failure [6]. However, the lack of successful long-term weight loss in overweight and obese individuals may be due to the format of the weight management programs, such that dietary and lifestyle recommendations alone may not be enough [8]. In fact, the high attrition rate and/or weight regain after initial weight loss is so common that many researchers have tried to address this key issue by also adding other components to the treatment or intervention [22]. For example cognitive behavioural therapy, group support sessions, frequent medical or clinical appointments with health professionals and dietary supplements are additional strategies that have been included in some weight management programs or trials [4, 7, 25]. Research indicates that a multifactorial approach is likely to be optimal in achieving clinically meaningful weight loss results [22].

Another factor that is frequently overlooked in identifying adequate treatment for overweight and obesity is cost effectiveness [26]. If the socioeconomically disadvantaged are some of the worst affected in the obesity epidemic, then some of the more expensive commercially available weight loss programs (such as Weight Watchers™) [7] or strategies will probably not be an option for them. However, the evidence suggests that some form of social support yields better weight loss results than ‘going it alone’ [6]. It has also been clearly established that individuals can expect better health outcomes if they are well supported in a social sense [27, 28]. However, many individuals attempting weight loss don’t always receive the required social support for a variety of reasons [28].

Advances in internet communication technology in recent years have added another vehicle for the delivery of health promotion material, including weight loss programs. According to recent survey data, 99 % of Australian households have internet access, 69 % of Australians use social media and 95 % of these social media users have a Facebook account [29]. Almost 99 % of the population is covered by a mobile cellular network and there are 102.8 mobile cellular subscriptions for every 100 Australians [30]. This offers health promoters the opportunity to deliver cost-effective weight management programs. Internet-mediated social networking [31] increases this potential, as studies have shown that social media can provide social support to members [32] by motivating and inspiring one another. Social media can also offer a medium for information sharing [33]. Being a part of an online social community undergoing lifestyle modifications may even assist individuals to be more accountable for their progress, and improve motivation further. Moreover, a review of the literature indicates that online health improvement programs often result in positive change [34–36]. A number of internet-based health intervention studies have utilised an interactive or social element, such as discussion boards or chat rooms, with many providing tailored feedback generated via mobile monitoring devices or health professionals [37, 38]. To date there have been few studies that have examined the effectiveness of using the social media platform (such Facebook) in the area of weight management, and none promoting dietary *and* physical changes *without* providing feedback other than the support derived from other study participants [37, 38].

### Aim of this study

The aim of this project is to measure weight loss and other outcome measures in overweight and obese individuals when a weight management program is delivered via social media, compared to the same program presented in written information only. The study will be undertaken over a period of twelve weeks, with a twelve-week follow-up thereafter. This trial will: i) determine whether incorporating social media into a weight management program will assist overweight and obese individuals to achieve greater, more sustainable improvements in weight loss and other outcome measures than following the same dietary and lifestyle recommendations in written form alone; ii) elucidate the particular aspects of social media that assist overweight and obese individuals to achieve the greater improvements in weight loss and other outcome measures.

### Summary of intervention

One hundred and twenty overweight and obese participants from the Perth community will be enrolled into the study, and randomly divided into three groups: two intervention groups and a control group. The two intervention groups will be instructed to follow the CSIRO TWD [17] weight management program for the twelve-week intervention period. One of the intervention groups will receive the diet via the social networking website Facebook and will be enrolled into a support network which is hosted via the Facebook site. The other group will receive the intervention in written form (pamphlet) alone. Both of the intervention groups will be supplied with pedometers (G Sensor 2025 Accelerometer, Walk with Attitude Australia) and set a target of 10,000 steps per day. The control group will follow the Australian Dietary Guidelines [39] as well as National Physical Activity Guidelines for Adults [16] as usual care. Participants will complete a series of questionnaires which will evaluate key psychological variables (see below) and attend Curtin University for assessment of body weight and other clinical outcome variables (outlined in the Design/Methods section) at weeks 0, 6, and 12 and at further 12-week follow-up (week24). The primary outcome measures in this trial include weight, body composition and waist circumference. Blood glucose, insulin, blood pressure, arterial stiffness [40], physical activity, eating behaviour, mental well-being (stress, anxiety, and depression), social support, self-control, self-efficacy, Facebook activity, and program evaluation are secondary outcome measures. Changes in psychological and clinical outcomes are expected to be greatest in the intervention group delivered and supported by social media relative to the pamphlet intervention and control groups. It is anticipated that social media will provide an invaluable resource for health professionals, serving as a low maintenance vehicle for communicating with patients and a source of social support and information sharing for individuals undergoing lifestyle modifications.

### Hypotheses

In the present study, it is hypothesised that, compared to the Control Group, the Pamphlet Group will experience moderate improvements in outcome measures (including weight loss of approximately 2 kg) as a result of the twelve-week pamphlet-delivered intervention. It is also hypothesised that, compared to the Control Group, the Facebook Group will experience greater improvements in outcome measures (including weight loss of approximately 8 kg) as a result of the twelve-week social media-delivered intervention. It is further hypothesised that participants in the Facebook Group will experience greater improvements in outcome measures compared to the Pamphlet Group due to the support they receive from using Facebook. Finally, it is hypothesised that at the expiration of the twelve-week intervention period the Facebook Group will be self-sustaining with respect to their ongoing stable weight.

## Methods/design

### Participants

A cohort of 120 overweight and obese individuals with a body mass index (BMI) between 25–40 kg/m^2^ and aged between 21 and 65 years will be recruited from the Perth community via flyers posted at community noticeboards, advertisements in local newspapers and advertisements on local community radio stations. Eligible participants will also be required to have access to a computer, laptop, tablet or Smartphone. Exclusion criteria will include smoking, lipid lowering medication, use of steroids and other agents that may influence lipid metabolism, use of warfarin, diabetes mellitus, hypo- or hyperthyroidism, cardiovascular events within the last 6 months, major systemic diseases, gastrointestinal problems, proteinuria, liver disease, renal failure, weight fluctuations over the past 6 months, vegetarianism or participation in any other clinical trials within the last 6 months. All participants will be required to provide written informed consent before the trial commences. All identifiable information from participants will be coded. This study will be conducted according to the ethical guidelines that are specified in the Curtin University Human Research Ethics Committee (HREC) and the National Health and Medical Research Council (NHMRC) guidelines. This trial has been approved by the Curtin University HREC (approval number: HR90/2014) and has been registered with ANZCTR (registration number: ACTRN12614000536662), on 21 May 2014.

### Study design

**Fig. 1:**
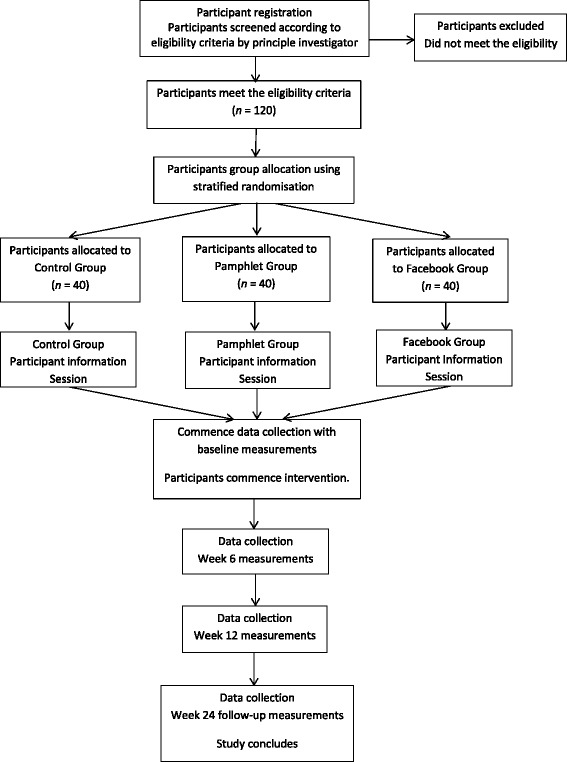
Flow of participants

This will be a three-armed, randomised, controlled, parallel design intervention study undertaken over a 12 week period, with a subsequent 12 week follow-up. Interested participants will be screened according to the inclusion/exclusion criteria and those eligible will be allocated a number, stratified according to age and gender, and then randomly allocated to one of the three groups of 40 participants, using dedicated computer randomisation software [41, 42] The allocated number will also be used as the participants’ identification number, to be used on all records and questionnaires. The three groups will consist of: the Control Group who will follow the Australian dietary guidelines [39] and National Physical Activity Guidelines for Adults [16], the Pamphlet Group who will be instructed in the weight management program by written information, and the social media group who will receive exactly the same weight management program via Facebook in the Facebook Group. [Please see Fig. 1: Flow of participants.] The initial twelve-week weight management program will be presented to the two intervention groups as a condensed version of the CSIRO TWD [17], which includes information of the weight management program, along with weekly checklists available from the CSIRO TWD [43]. Before the commencement of the trial, participants will attend an information session specific to their group allocation, where the participation requirements, including questionnaires and outcome measurements, will be explained. An additional file explains this in greater detail [please see Additional file 1]. During the Facebook Group information session, participants will be provided with additional information about using Facebook in this context. Facebook Group participants will also be made aware of the role of the primary investigator as administrator and facilitator of the Facebook Group. In addition, participants in this group will be informed that at the completion of the twelve-week intervention period, all facilitation will cease, however the group will still be monitored by the facilitator to ensure that they continue to conduct themselves according the instructions provided. This change in strategy for the twelve-week follow-up period will be used to determine whether the Facebook Group has become self-sustaining. An additional file explains the added information provided for Facebook Group participants in greater detail [please see Additional file 2].

### Outcome measures

The primary outcome measures for this study is weight loss, body composition and waist circumference. Secondary outcome measures include blood glucose, insulin and lipids, blood pressure, arterial stiffness and hip circumference, as indicators of changes to cardiometabolic disease risk factors. Dietary and physical activity compliance, eating behaviour, quality of life, mental well-being (stress, anxiety, and depression), self-control, self-efficacy, Facebook activity, and program evaluation will also be evaluated as further secondary outcome measures. These clinical and self-reported measurements have been chosen to test the participants’ adherence to the weight management program, and to compare the outcomes of the two different weight loss program delivery methods.

### Assessments

All participants will be required to attend regular clinical appointments for a duration of approximately 15 min, as follows: at baseline, at weeks 6 and 12, and then again for a follow-up appointment at week 24. Prior to each appointment, participants will complete a Three-Day Food Record and selection of questionnaires to monitor compliance and address some of the secondary outcome measures. The Bouchard Three-Day Physical Activity Record [44] will be used to measure of total physical activity. The Three-Factor Eating Questionnaire (TFEQ) [45] will provide a measure of dietary restraint, disinhibition and hunger, and will also be used here to assess changes in satiety. The Self-Efficacy Scale [46] will be used to assess changes to participants’ self-efficacy. The WHO Quality of Life Questionnaire [47] will be used to determine changes in participants’ quality of life. The short version of the Depression Anxiety Stress Scale (DASS 21) [48] will be used to measure changes in general psychological wellbeing. The Self-Control (Brief) Scale [49] will be used to provide an understanding of participants’ impulse control as it relates to eating behaviour. The Diet and Physical Activity Survey, constructed using the Theory of Planned Behaviour [50], will be used to determine participants’ intentions with regard to the dietary and physical activity guidelines being used in the trial. The Survey of Weight Management Program has some general questions regarding the ease of use of the dietary and physical activity guidelines being utilized; there will be an extra section for Facebook Group participants to assess the preferred device for accessing the weight management information provided on Facebook. For Facebook Group participants only, the Social Media Survey will be used to assess participants’ attitudes to social media. The final questionnaire will also be administered to Facebook Group participants only; the Facebook Intensity and Network Density Scales contains a combination of questions used in previous social media research [51, 52]. It will be adapted for this study to assess the degree to which participants make use of Facebook, as well as the strength and frequency of the social interactions within the Facebook Group. In addition, the Facebook Group page will be printed at the end of each week to corroborate self-reported Facebook usage data and monitor participants’ online behaviour. Participants will be given questionnaires (including the Food Record and Physical Activity Record) at each clinic appointment, according to their group allocation. [For further information regarding the assessments please see Table 1: Schedule of Assessments.] Participants will be instructed to use their participant identification number only when completing all documents relating to this trial [42].

**Table 1:** Schedule of assessments

Measurement by Group	Week 0			Week 6			Week 12			Week 24		
	CG	PG	FG	CG	PG	FG	CG	PG	FG	CG	PG	FG
3-Day Food Record	•	•	•	•	•	•	•	•	•	•	•	•
Physical Activity Record	•	•	•	•	•	•	•	•	•	•	•	•
Three Factor Eating Questionnaire	•	•	•	•	•	•	•	•	•	•	•	•
Self-Efficacy Scale	•	•	•	•	•	•	•	•	•	•	•	•
WHO Quality of Life	•	•	•	•	•	•	•	•	•	•	•	•
Depression Anxiety Stress Scale	•	•	•	•	•	•	•	•	•	•	•	•
Self-Control (Brief) Scale	•	•	•	•	•	•	•	•	•	•	•	•
Diet & Physical Activity Survey	•	•	•	•	•	•	•	•	•	•	•	•
Survey of Weight Management Program*				•	•	•	•	•	•			
Social Media Survey			•			•			•			•
Facebook intensity & Network Density Scales						•			•			•
Height	•	•	•									
Weight	•	•	•	•	•	•	•	•	•	•	•	•
Blood pressure	•	•	•	•	•	•	•	•	•	•	•	•
Arterial stiffness	•	•	•	•	•	•	•	•	•	•	•	•
Blood glucose	•	•	•	•	•	•	•	•	•	•	•	•
Waist circumference	•	•	•	•	•	•	•	•	•	•	•	•
Hip circumference	•	•	•	•	•	•	•	•	•	•	•	•
Body Composition	•	•	•	•	•	•	•	•	•	•	•	•
Blood lipids	•	•	•				•	•	•			
Blood insulin	•	•	•				•	•	•			

Key: CG = Control Group; PG = Pamphlet Group; FG = Facebook Group

* There will be two versions of the Survey of Weight Management Program. Version 2 will include an additional section specific to Facebook Group participants only

At each clinical appointment, weight will be recorded in light clothing without shoes. (UM-018 Digital Scales; Tanita Corporation, Tokyo, Japan). Body composition will be measured by bioelectrical impedance analysis (BIA) in light clothing without shoes using the digital scales just mentioned. Height will be measured without shoes to the nearest 0.1 cm using a wall-mounted stadiometer (26SM 200 cm SECA, Hamburg, Germany). Height and weight measurements will be used to calculate participants’ BMI. Waist circumference will be measured in the standing position at the narrowest area between the lateral lower rib and the iliac crest; hip circumference will be measured at the widest area across the buttocks. Briefly, fasting blood glucose will be taken using the Accu-Chek® Performa glucometer and lancing device (Roche, Australia). Blood pressure will be assessed with an automated, calibrated sphygmomanometer (Dinamap, Compact T, Critikon, Germany). Pulse contour analysis (PCA) will be used to assess arterial stiffness using the Pulse Trace PCA 2 (CareFusion, NSW, Australia). In addition, at weeks 0 and 12 participants will be required to attend a local pathology collection centre (PathWest Laboratory Medicine, Western Australia) for blood tests to measure lipids (triglycerides (TG), total cholesterol (TC), low density lipoprotein (LDL) and high density lipoprotein (HDL)) and insulin. [For further information regarding the assessments please see Table 1: Schedule of Assessments.]

### Statistical analysis

Based on a three group study with repeated measures, a sample size of 96 achieves 80 % statistical power to detect a medium to large effect size (Cohen’s *d* = 0.4); that is, a difference of 6 kg between the two intervention groups, with alpha set at 0.05. To allow for an attrition rate of 20 %, a total of 120 participants will be recruited; i.e. 40 participants per group. Stratified randomisation will be used to ensure that each group is matched in age and gender. The results will be analysed using a mixed repeated measures analysis of variance (ANOVA) design. Data will be expressed as mean (±SEM) and assessed for normality to ensure that the assumptions of the analysis are met. If significant between groups effects are present, post hoc analysis will be conducted using the Least Significant Difference (LSD) method. Statistical significance will be considered at p < 0.05. All statistical analyses will be performed using SPSS 21.0 for Windows (SPSS Inc., Chicago, IL), and conducted using the participant identification number only [42].

## Discussion

The purpose of this study is to evaluate the use of social media as a health promotion tool, specifically to promote dietary and physical activity changes for overweight and obese individuals, without providing any form of tailored feedback. The principle investigator, in the role of Facebook Group facilitator, will initiate discussion within the group by posting general questions or comments to the Facebook Group’s wall, but will not respond to participants’ comments or questions. This type of facilitation will be used to ‘break the ice’ and encourage group members to interact with each other. In the best case scenario, it is anticipated that participants will then view other group members as a source of social support, as they all have weight loss as a common goal and quite possibly may have experienced similar issues with regards to being overweight. The benefits of this type of support (provided by a discreet, interactive online social group) include being able to freely share their views and information, ask and answer questions and ask for and offer assistance. *Providing support to others* may be as beneficial to the individual as receiving support [53, 54]. Other possible social-related benefits may come in the form of encouragement (to persevere with the program), the formation of walking groups (to meet the physical activity requirements) and praise for the achievement of any weight loss milestones. Another important advantage conferred by membership of a group of this nature is the potential for clarification of the elements of the weight management program. For example, if a group member experiences any difficulty integrating the weight management guidelines into their daily life, they may find help can be obtained from other group members more experienced in food preparation and deciphering amounts (or units) of foods or ingredients, *without the need to consult a health professional*. This type of support (assistance and understanding, given and received) may augment the weight loss expected when following a proven weight management program, as help will potentially be available from other group members. Indeed, participants in the original trials of the CSIRO TWD received intensive dietetic support [11, 20, 55], so it is hoped that the social support available within the Facebook Group will provide a different, but effective substitute. Further, it is speculated that if Facebook Group participants take full advantage of the social support built into this intervention, then long term weight maintenance will also be more feasible for them.

If using social media to assist weight loss is found to be effective then health promotion professionals, policy-makers and groups interested in changing behaviour may be encouraged to adopt social media as an additional health promotion tool. Using social media for health promotion could have many advantages, such as being able to reach large groups wherever the technological infrastructure is in place. These individuals can then access the information and updates provided in their own time and place. It has the potential to provide health care and health promotion professionals the ability to manage a large and geographically disparate case-load, with a minimal investment of time, and may provide a future role for health professionals. These findings may also have clinical applications, allowing health care professionals to generate support networks for their patients undergoing diet and lifestyle modification, especially for the socially or geographically isolated, or those whose access to health care workers is limited or infrequent. Other advantages include the fact that social media is essentially free to use, so this tool would also be cost-effective and therefore potentially more readily adopted in real world settings than more expensive interventions that have been tested in other studies. Due to the interactive nature of the technology, feedback can also be collected from participating individuals. Moreover, there is the possibility of conducting focus groups with those who would be otherwise out-of-reach. It should not be inferred however, that social media should be utilised to completely replace in-person consultations with health professionals; rather, its most appropriate use is likely to be in providing on-going support for patients between appointments with relevant health professionals.

So far, studies examining the efficacy of using the social media platform to promote dietary and physical activity changes for weight management have been few. Aside from adapting assessments used in traditional health promotion interventions, methods of evaluating the potential health benefits of social media are still under development. It is therefore anticipated that the results of this study will add significantly to the body of knowledge regarding health promotion methods for weight management programs.

## Additional files

Additional file 1:
**Participant information sessions.**
Additional file 2:
**Weight management program within the Facebook Group.**

## Abbreviations

ANOVA: Analysis of variance
ANZCTR: Australian New Zealand clinical trial registry
BIA: Bioelectrical impedance analysis
BMI: Body mass index
CSIRO: Commonwealth scientific and industrial research organisation
DASS 21: Depression, anxiety, and stress scale, short version
HDL: High density lipoprotein
HREC: Human research ethics committee
IPAQ: International physical activity Questionnaire
LDL: Low density lipoprotein
NHMRC: National health and medical research council
PCA: Pulse contour analysis
TC: Total cholesterol
TFEQ: Three-factor eating questionnaire
TG: triglycerides
TWD: Total wellbeing diet
WHO: World health organisation

## References

[1] Chapman C (2012). Lifestyle determinants of the drive to eat: a meta-analysis. Am J Clin Nutr.

[2] Grundy SM (2004). Obesity, metabolic syndrome, and cardiovascular disease. J Clin Endocrinol Metabol.

[3] Jequier E (2002). Pathways to obesity. Int J Obes Relat Metab Disord.

[4] Wilborn C, Beckham J, Campbell B, Harvey T, Galbreath M, La Bounty P (2005). Obesity: prevalence, theories, medical consequences, management, and research directions. J Int Soc Sports Nutr.

[5] Sikorski C, Luppa M, Kaiser M, Glaesmer H, Schomerus G, König H (2011). The stigma of obesity in the general public and its implications for public health - a systematic review. BMC Public Health.

[6] Greener J, Douglas F, van Teijlingen E (2010). More of the same? Conflicting perspectives of obesity causation and intervention amongst overweight people, health professionals and policy makers. Soc Sci Med.

[7] Webb VL, Wadden TA, Tsai AG, Thomas FC (2012). Weight-loss programs: commercial and popular diets. Encyclopedia of Body Image and Human Appearance.

[8] Shaw KA, O’Rourke P, Del Mar C, Kenardy J. Psychological interventions for overweight or obesity. The Cochrane Database of Systematic Reviews. 2009;2005(2) 10.1002/14651858.CD003818.pub2 15846683

[9] Wycherley TP, Moran LJ, Clifton PM, Noakes M, Brinkworth GD (2012). Effects of energy-restricted high-protein, low-fat compared with standard-protein, low-fat diets: a meta-analysis of randomized controlled trials. Am J Clin Nutr.

[10] Skov AR, Toubro S, Rønn B, Holm L, Astrup A (1999). Randomized trial on protein vs carbohydrate in ad libitum fat reduced diet for the treatment of obesity. Int J Obes (Lond).

[11] Noakes M, Keogh JB, Foster PR, Clifton PM (2005). Effect of an energy-restricted, high-protein, low-fat diet relative to a conventional high-carbohydrate, low-fat diet on weight loss, body composition, nutritional status, and markers of cardiovascular health in obese women. Am J Clin Nutr.

[12] Lejeune MPGM, Kovacs M, Westerp-Plantenga MS (2005). Additional protein intake limits weight regain after weight loss in humans. Br J Nutr.

[13] McConnon A, Horgan GW, Lawton C, Stubbs J, Shepherd R, Astrup A, et al. Experience and acceptability of diets of varying protein content and glycemic index in an obese cohort: results from the Diogenes trial. Eur J Clin Nutr. 2013;1–6. 10.1038/ejcn.2013.99 23778783

[14] Ho S, Dhaliwal S, Hills A, Pal S (2011). Acute exercise improves postprandial cardiovascular risk factors in overweight and obese individuals. Atherosclerosis.

[15] Pal S, Radavelli-Bagatini S, Ho S (2013). Potential benefits of exercise on blood pressure and vascular function. J Am Soc Hypertens.

[16] Department of Health and Ageing: National Physical Activity Guidelines for Adults. In. Edited by Department of Health and Ageing; Canberra, Australia: Australian Government; 2005

[17] Noakes M, Clifton P (2006). The CSIRO Total Wellbeing Diet Book 2.

[18] National Health and Medical Research Council: Nutrient reference values for Australia and New Zealand. In*.* Edited by National Health and Medical Research Council; Canberra, Australia: Australian Government; 2005.

[19] Larsen T, Larsen S-M, Dalskov M, van Baak S, Jebb A, Pfeiffer JA (2010). Diets with high or low protein content and glycemic index for weight-loss maintenance. N Engl J Med.

[20] Wycherley TP, Brinkworth GD, Clifton PM, Noakes M (2012). Comparison of the effects of 52 weeks weight loss with either a high-protein or high-carbohydrate diet on body composition and cardiometabolic risk factors in overweight and obese males. Nutrition and Diabetes.

[21] Pal S, Cheng C, Ho S (2011). The effect of two different health messages on physical activity levels and health in sedentary overweight, middle-aged women. BMC Public Health.

[22] Wyld B, Harrison A, Noakes M (2010). The CSIRO Total Wellbeing Diet Book 1: sociodemographic differences and impact on weight loss and well-being in Australia. Public Health Nutr.

[23] Donnelly J, Donnelly S, Blair J, Jakicic M, Manore J, Rankin B (2009). Appropriate physical activity intervention strategies for weight loss and prevention of weight regain for adults. Med Sci Sports Exerc.

[24] Bautista-Castan˜o I, Molina-Cabrillana J, Montoya-Alonso JA, Serra-Majem L. Variables predictive of adherence to diet and physical activity recommendations in the treatment of obesity and overweight, in a group of Spanish subjects. Int J Obes. 2004;28:697–705. 10.1038/sj.ijo.0802602 14993911

[25] Egger G, Pearson S, Pal S, Swinburn B (2007). Dissecting obesogenic behaviours: the development and application of a test battery for targeting prescription for weight loss. Obes Rev.

[26] Byrne NM, Meerkin JD, Laukkanen R, Ross R, Fogelholm M, Hills AP (2006). Weight loss strategies for obese adults: personalized weight management program vs standard care. Obesity.

[27] Kumar S, Kumar R, Calvo M, Avendano K, Sivaramakrishnan L (2012). Social support, volunteering and health around the world: Cross-national evidence from 139 countries. Soc Sci Med.

[28] Grant N, Hamer M, Steptoe A (2009). Social isolation and stress-related cardiovascular, lipid, and cortisol responses. Ann Behav Med.

[29] Pages Y. Yellow™ social media report. What Australian people and businesses are doing with social media. In*.* Edited by Holmes D, Brough K. Melbourne, Australia; Yellow Pages; 2014.

[30] ACMA (2011). The internet service market and Australians in the online environment.

[31] ABS (2011). Household use of information technology, Australia 2010–11.

[32] Liu CY, Yu CP (2013). Can facebook use induce well-being?. Cyberpsychol Behav Soc Netw.

[33] Steinfield C, Ellison NB, Lampe C (2008). Social capital, self-esteem, and use of online social network sites: A longitudinal analysis. J Appl Dev Psychol.

[34] Korda H, Itani Z (2013). Harnessing social media for health promotion and behavior change. Health Promot Pract.

[35] Arem H, Irwin M (2011). A review of web-based weight loss interventions in adults. Obes Rev.

[36] Carr LJ, Bartee RT, Dorozynski C, Broomfield JF, Smith ML, Smith DT (2008). Internet-delivered behavior change program increases physical activity and improves cardiometabolic disease risk factors in sedentary adults: Results of a randomized controlled trial. Prev Med.

[37] Ashrafian H, Toma T, Harling L, Kerr K, Athanasiou T, Darzi A (2014). Social networking strategies that Aim to reduce obesity have achieved significant although modest results. Health Aff.

[38] Williams G, Hamm MP, Shulhan J, Vandermeer B, Hartling L (2014). Social media interventions for diet and exercise behaviours: a systematic review and meta-analysis of randomised controlled trials. BMJ open.

[39] National Health and Medical Research Council: Eat for health. Australian dietary guidelines. In*.* Edited by National Health and Medical Research Council; Canberra, Australia: Australian Government; 2013.

[40] Pal S, Radavelli-Bagatini S (2013). Association of arterial stiffness with obesity in Australian women: a pilot study. J Clin Hypertens.

[41] Research Randomizer (Version 4.0) [Computer software] [http://www.randomizer.org/]

[42] Schulz KF, Altman DG, Moher D (2010). CONSORT 2010 Statement: Updated guidelines for reporting parallel group randomised trials. J Clin Epidemiol.

[43] Noakes M, Clifton P. The CSIRO Total Wellbing Diet Melbourne, Australia: Penguin; 2005.

[44] Bouchard C. Bouchard Three-Day Physical Activity Record. In Medicine & Science in Sports & Exercise A Collection of Physical Activity Questionnaires for Health-Related Research 1997;29(6):19–24. 9243481

[45] Stunkard AJ, Messick S (1985). The Three-Factor Eating Questionnaire to measure dietary restraint, disinhibition and hunger. Journal of Psychosomolic Research.

[46] Schwarzer R, BaBler J, Kwiatek P, Schroder K, Zhang JX (1997). The assessment of optimistic self-beliefs: comparison of the German, Spanish, and Chinese versions of the general self-efficacy scale. Appl Psychol Int Rev.

[47] World Health Organisation: WHO Quality of Life - Bref. Introduction, administration, scoring and generic version of the assessment; Department of Mental Health; Geneva, Switzerland; WHO; 1996.

[48] Antony MA, Bieling PJ, Cox BJ, Enns MW, Swinson RP (1998). Psychometric properties of the 42-item and 21-item versions of the depression anxiety stress scales in clinical groups and a community sample. Psychol Assess.

[49] Tangney JP, Baumeister RF, Boone AL (2004). High self-control predicts good adjustment, less pathology, better grades, and interpersonal success. J Pers.

[50] Constructing a Theory of Planned Behavior Questionnaire [http://people.umass.edu/aizen/pdf/tpb.measurement.pdf]

[51] Zhao J, Ha S, Widdows R (2013). Building trusting relationships in online health communities. Cyberpsychol Behav Soc Netw.

[52] Ellison NB, Steinfield C, Lampe C (2007). The benefits of Facebook “friends:” social capital and college students’ use of online social network sites. Journal of Computer-Mediated Communication.

[53] Verheijden MW, Bakx JC, van W, Koelen MA, van Staveren WA (2005). Role of social support in lifestyle-focused weight management interventions. Eur J Clin Nutr.

[54] Riessman F (1990). Restructuring help: A human services paradigm for the 1990s. Am J Community Psychol.

[55] Keogh JB, Brinkworth GD, Clifton PM (2007). Effects of weight loss on a low-carbohydrate diet on flow-mediated dilatation, adhesion molecules and adiponectin. Br J Nutr.

