# Supplementary material for: Using new technologies to promote weight management: a randomised controlled trial study protocol
Source: BMC Public Health. 2015 May 27;15:509. doi: 10.1186/s12889-015-1849-4 (PMC4445522; doi:10.1186/s12889-015-1849-4)
Supplement: Additional file 1: — Participant information sessions. [file 12889_2015_1849_MOESM1_ESM.docx]

**Additional file 1: Participant information sessions**

Participant information sessions will be held after recruitment, and consist of three separate information sessions for the different groups, as follows:

*The Control Group*

Participants in the Control Group will be instructed to follow the Australian Dietary Guidelines together with the National Physical Activity Guidelines for Adults [as mentioned in the Methods/Design section of the study protocol]. Also included in this session will be the schedule of clinic and pathology appointments, including the outcome measurements collected, the instructions for a completing the Three-Day Food Record, the Three-Day Physical Activity Record, as well as the questionnaires. The dietary and physical activity guidelines will be given to participants at the end of their first appointment. This is to ensure that participants do not begin the intervention before baseline measurements are collected.

*The Pamphlet Group*

Participants in the Pamphlet Group will be instructed to follow the CSIRO Total Wellbeing Diet, which they will receive in booklet form along with a weekly checklist [as mentioned in the Methods/Design section of this study protocol]. Also explained at this session will be the schedule of clinic and pathology appointments, including the outcome measurements collected, the instructions for a completing the Three-Day Food Record, the Three-Day Physical Activity Record, as well as the questionnaires. Participants will be supplied pedometers at this session; however participants will be given the booklet at the end of their first appointment. This is to ensure that participants do not begin the intervention before baseline measurements are collected.

*The Facebook Group*

The Facebook Group will be instructed to follow The CSIRO Total Wellbeing Diet, which will be posted to a dedicated group on Facebook, with the privacy settings set to ‘secret’, and will consist of snapshots of all of the information from the booklet given to the Pamphlet Group, so that both the Facebook Group and the Pamphlet Group receive identical information, as well as given the weekly checklists [as mentioned in the Methods/Design section of this study protocol]. Also explained at this session will be the schedule of clinic and pathology appointments, including the outcome measurements collected, the instructions for a completing the Three-Day Food Record, the Three-Day Physical Activity Record as well as the questionnaires. Participants will be supplied pedometers at this session; however participants will be added to the group on Facebook after of their first appointment. This is to ensure that participants do not begin the intervention before baseline measurements are collected.

In addition, Facebook Group participants will be shown how to use the Facebook Group, as well as how to interact with other group members (for example sharing recipes, how they are integrating the program in the daily lives, forming walking groups, inspiring and motivating other, posting before and after pic of themselves, congratulating others for their successes, problem solving etc), and will then be invited to join the closed Facebook Group.

*All groups*

At the conclusion of each participant information session, participants will be invited to ask questions, or raise any concerns that they may have.
