# Supplementary material for: Using new technologies to promote weight management: a randomised controlled trial study protocol
Source: BMC Public Health. 2015 May 27;15:509. doi: 10.1186/s12889-015-1849-4 (PMC4445522; doi:10.1186/s12889-015-1849-4)
Supplement: Additional file 2: — Weight management program within the Facebook Group. [file 12889_2015_1849_MOESM2_ESM.docx]

**Additional file 2: Weight management program within the** **Facebook Group**

The principle investigator will be the administrator and facilitator of the Facebook Group. The group facilitator will spend two hours per week monitoring the Facebook site. Participants will be advised that the facilitator will have a presence within the site, and will monitor posts to makes sure that they fall within the guidelines specified at the Participant Information Session and stated in hard copy on the Participant Information Sheet.

After all of the participants have joined the Facebook Group, snapshots of the entire weight management program used in the booklet will be posted to the Facebook Group as photos in a series of photo albums. This will save participants the trouble of scrolling through the more recent posts and comments, added over time, to review the program when needed. Being able to easily access the program also enables participants in this group the same advantage as the Pamphlet Group, who are able to read and review the program in hard copy at their leisure.

To stimulate discussion between Facebook Group members a list of general questions will be created. Twice a week, the facilitator will post one of the questions from the list to the group’s wall. Examples of the types of questions to be posted include: Does anyone have a favourite recipe based on the program that they would like to share with the group? And, what is a good way to incorporate thirty minutes of moderate physical activity into the day? Without directly telling the participant what to do on the Facebook site, it is hoped that this minimal facilitation will help spontaneous discussions begin to take place during the course of the twelve-week intervention period as well.

Participants using Facebook will be instructed to be polite in their interactions with other members, and never to say anything that may embarrass other members, or clutter up the newsfeed with trivial information like “Sitting down for my morning coffee”. They will be instructed to post their thoughts on the weight management program and how they are integrating it into their lives, any achievements brought about by using the program, or ask for assistance from group members if they encounter a problem. Participants in this group will be instructed to offer praise and encouragement for the achievements of others, offer useful tips, form walking groups, post recipes or motivational quotes. It will be made extremely clear to the participants using Facebook that this group is in place to provide a friendly and supportive environment for all members.

The Facebook Group facilitator will also monitor the comments posted by the group daily, and will act on any negative comments or erroneous statements posted on the group’s wall with an appropriate response or correction. In the event of an offensive comment, the facilitator will remove the post immediately, and contact the offending participant privately. If the issue cannot be resolved or there is a repetition of the offence, the offending participant will be removed from the group, and will take no further part in this project.

*Privacy*

The Facebook Group will be set as a ‘Secret Group’, whereby only group members can see the group, who’s in it and what members post. In addition, the group’s administrator (principle investigator) will be the only member to grant approval to new members. (These settings are features provided by Facebook.) These precautions will be taken to provide a discreet online environment for the participants in the Facebook Group, and also to ensure that no-one else can see or join this group.
